# Apolipoprotein C-I Polymorphism and Its Association with Serum Lipid Levels and Longevity in the Bama Population

**DOI:** 10.3390/ijerph14050505

**Published:** 2017-05-09

**Authors:** You Li, Yongquan Huang, Xue Liang, Bingshuang Long, Shiyi Chen, Jiahao Lian, Yi Wei, Zhiyong Zhang, Jian Qin

**Affiliations:** 1 Department of Occupational and Environmental Health, Faculty of Public Health, Dali University, Dali 671000, China; liyou121300@163.com; 2 Department of Occupational and Environmental Health, School of Public Health, Guangxi Medical University, Shuangyong Road No.22, Nanning 530021, China; huangyongquan669@163.com (Y.H.); 490275305@139.com (X.L.); 15677188715@163.com (B.L.); chensy1016@163.com (S.C.); 18778684045@163.com (J.L.); weiyi1103wy@163.com (Y.W.)

**Keywords:** apolipoprotein C-I (ApoC-I), genetic polymorphisms, longevity, blood lipids

## Abstract

This study aims to determine the association between the apolipoprotein C-I polymorphism and the longevity and genetic variants in ApoC-I that can influence the serum lipid levels in Bama. ApoC-I genotypes were determined by Taqman single nucleotide polymorphism (SNP) genotyping assays in 178 long-lived inhabitants (longevity group aged from 90 to 110 years), 147 healthy controls (Control 1 group aged from 40 to 79 years old) from Bama County, and 190 healthy controls (Control 2 group aged from 40 to 79 years old) from Nandan County without a family history of longevity. Statistical analysis was conducted using SPSS 16.0. All genotype distributions of rs584007 and rs4420638 were consistent with the Hardy–Weinberg equilibrium (*p* > 0.05). Significant differences were observed in the frequencies of the three genotypes (GG, AG, and AA) among the longevity and the two control groups (χ^2^ = 11.238, *p* = 0.024) for rs584007. No significant differences were observed in the frequencies of the three genotypes (GG, AG, and AA) among the longevity and the two control groups (χ^2^ = 4.587, *p* = 0.318) for rs4420638. The levels of total cholesterol (TC), triglycerides (TG), high-density lipoprotein-cholesterol (HDL-c), and low-density lipoprotein-cholesterol (LDL-c) were not different among the three genotypes of rs584007 in the three groups. The levels of HDL-c for GG, AG, and AA were significantly different (the highest being in the longevity group), while the levels of TG for AA and AG genotypes (the lowest being in the longevity group) and the levels of LDL-c for AG were significantly different (*p* < 0.05) among the three groups for rs584007. The levels of TG and HDL-c were significantly different among the three rs4420638 genotypes in the longevity group. The levels of TC for GG, AG, and AA were significantly different in the Control 2 group, while the levels of TG and HDL-c for AA and AG genotypes were significantly different (*p* < 0.05) among the three groups for rs4420638. The level of HDL-c was highest in the longevity group for AA and AG genotypes, and the level of TG was highest in the Control 2 group for rs4420638. Serum lipid parameters were related to environmental factors, including age, gender, BMI, DBP, SBP, rs4420638, and rs584007. The ApoC-I polymorphism might be one of the genetic factors of longevity in Bama. The ApoC-I rs4420638 and rs584007 SNPs are associated with serum TG and HDL-c levels in the longevous population.

## 1. Introduction

Apolipoprotein C-I (ApoC-I) is a member of the apolipoprotein family, which includes ApoC-I, ApoC-II, and ApoC-III, low-molecular-weight lipoprotein components. The human ApoC-I and ApoE genes are closely connected in a 45-kilobase (kb) region of chromosome 19 [1,2]. ApoC-I, a constituent of triglyceride-rich lipoproteins, is involved as a cofactor in enzymatic reactions of lipid metabolism with high-density lipoproteins (HDLs) [3].

ApoC-I is involved in the maintenance of HDL structure, regulation of lipase enzymes [4,5], and inhibition of the absorption of triglyceride (TG)-rich lipoproteins through hepatic receptors, especially low-density lipoprotein (LDL) receptor-related protein [3,6]. Moreover, ApoC-I cooperated with ApoE takes part in several biological processes, such as cholesterol metabolism, membrane reconstitution, neuronal apoptosis, and recombination [7]. ApoC-I is in connection with a hyperlipidemic condition [8], Alzheimer’s disease (AD) [9], cardioprotection, cancer cell proliferation [10], and metabolic syndrome [11]. Apart from the aforementioned diseases, ApoC-I is also involved in ageing and longevity [12]. In addition, studies have indicated that dyslipidemia has been a significant risk factor for coronary heart disease (CHD), which might contribute to human ageing and longevity [13,14].

Longevity and ageing are a complex process that results from the interaction between environmental and multiple genetic factors [15], which can regulate both cellular and metabolic functions, and the concentrations of apolipoproteins and lipoproteins [16,17]. A study on twins has shown that human genetic factors determine 15–30% of longevity traits [18]. Meanwhile, the heritability evaluation of lipoproteins and apolipoproteins between the twin and family studies is 40–80% [19,20], indicating a considerable genetic contribution. ApoC is one of the known longevity genes, which also includes other genes, such as ApoE, GSTT1, IL-6, IL-10, SIRT6, and FOXO3a [21,22]. The related reports on the association between the gene polymorphisms of Apos and longevity have mainly involved apoA, apoB, apoE, and apoC [23]. ApoC-I induces cardioprotection and regulates lipid metabolism through the modulation of Δψm and oxidative phosphorylation resulting in longevity often being spared from age-related diseases, especially cardiovascular disease (CVD), AD, diabetes mellitus, and cancer [24].

The population of Bama County is located in the Hongshuihe River Basin of Guangxi Province, having become well-known to the world as the longevity village, as the group has had little genetic diversity in the past few decades [25,26].

The association between the ApoC-I polymorphism and the risk of AD in humans has been studied previously [27], but the relationship between the ApoC-I polymorphism and the plasma or serum lipid levels in longevity participants has not been reported. In this study, we examine the relationship between the ApoC-I polymorphism and the serum lipid profiles in longevity and control populations to further explore the longevity of the population in Bama.

## 2. Methods

### 2.1. Study Population

In our study, 178 so-called “longevity” subjects (127 females and 51 males, age 94.30 (4.21) years [mean (SE)], range 90–108 years) were recruited to participate in the study. Longevity was defined as living to 90 years of age or older. The Control 1 group consisted of 68 females and 79 males (age 65.14 [11.26] years, range 40–79 years) from Bama County (environment fit). There were 190 individuals included in the Control 2 group (74 males and 116 females, age 53.98 [10.51] years, range 40–79 years) from Nandan County, which is about 160 km away from Bama County. We selected the town in Nandan County, whose economic income level was similar to that of Bama County, as the external control area (environmentally unmatched). There were no long-lived family members in either control group. Long-lived family members had to meet the following conditions: (1) aged 90 or older, and (2) having one or more living brother or sister who satisfied the first criterion. The ages of the participants were defined officially by their identity card or residence registration booklet and the accounts of their offspring and other important sociographic events. All subjects were healthy and there was no evidence of related-diseases such as atherosclerosis, CHD (coronary heart disease), and diabetes. The participants did not take medications that might affect serum lipid levels (for example, statins or fibrates, beta-blockers, diuretics, or hormones). The study was reviewed and approved by the ethics committee of Guangxi Medical University (Project Identification Code: 201503010-2). All participants provided written informed consent.

### 2.2. Biochemical Analysis

A venous blood sample of 5 mL was obtained from each participant who had previously fasted overnight. About 3 mL of blood sample was used to determine serum lipid levels. The levels of serum TC, TG, HDL-c, and LDL-c were determined by standard enzymatic methods with commercially available kits.

### 2.3. Genotyping

The remaining 2 mL blood sample was used to extract genomic DNA by the Chelex-100 method [28]. The extracted DNA was stored at −20 °C until analysis. We used the Haploview4.2 package (according to *r*^2^ ≥ 0.80 and MAF ≥ 5%) and a website of gene function prediction (http://manticore.niehs.nih.gov/snpfunc.htm) as well as literature reports to select the loci located in the functional area. PCR was performed according to the standard methods. The reaction’s mixture (total of 10 μL) included with 1 μL of genomic DNA, 0.25 μL of Assay-on-Demand SNP Genotyping Assay Mix (40×) (Applied Biosystems Co., Ltd., Waltham, MA, USA), 3.75 μL of ddH_2_O, and 5 μL of TaqMan Universal PCR Master Mix. Each PCR cycle consisted of the following conditions: predenaturation for 10 min at 95 °C, followed by 40 cycles of denaturation for 15 s at 92 °C, annealing for 1 min at 60 °C, and extension for 60 s at 72 °C. The fluorescence intensity of the two different dyes was tested to obtain the allelic discrimination plot and distinguish individual genotypes (SDS 2.3 software, Applied Biosystems, Waltham, MA, USA) with PCR.

### 2.4. Statistical Analysis

All statistical tests were carried out using SPSS 16.0 (SPSS Inc., Chicago, IL, USA). Quantitative variables were expressed as mean ± SDs. An analysis of variance (ANOVA) was used to compare quantitative variables, and the chi-square test was performed to compare categorical variables. Genotype frequencies of all SNP were found to be in Hardy–Weinberg equilibrium. The chi-square test was used to compare genotype and allele frequency differences among the groups. A Bonferroni correction was applied to determine the proper level of statistical significance (*p* = 0.05/number of comparisons, number of comparisons = 3, *p* < 0.017). The association of ApoC-I genotypes and serum lipid levels was evaluated by analysis of co-variance (ANCOVA). To evaluate the association between the ApoC-I polymorphism and serum lipid levels or several environmental factors, multiple linear regression analysis was performed. *p*-values less than 0.05 on a two-sided test were considered statistically significant.

## 3. Results

### 3.1. General Characteristics and Serum Lipid Levels

The demographic and biochemical characteristics among three groups were shown in Table 1. The mean ages of the three groups were 94.30 ± 4.21 (range from 90 to 108 years old), 65.14 ± 11.26 (range from 40 to 79 years old), and 53.98 ± 10.51 (range from 40 to 79 years old) years, respectively. The BMI and the level of TG were lower in the longevity group than those in the two control groups, while serum concentrations of LDL-c and HDL-c and the level of SBP in the longevity group were higher than those in the two control groups (*p* < 0.01).

### 3.2. Hardy Weinberg Equilibrium Test of the Different Populations

The chi-square test revealed that all genotype distributions were consistent with Hardy–Weinberg equilibrium (*p* > 0.05) (Table 2).

### 3.3. Genotypic and Allelic Frequencies

AG was the dominant genotype in all participants, with a frequency of 0.52 for rs584007 (Table 3). We observed significant differences in the frequencies of the three genotypes (GG, AG, and AA) among the longevity and two control groups (χ^2^ = 11.238, *p* = 0.024) for rs584007. AA was the dominant genotype in all participants, with a frequency of 0.802 for rs4420638. There were no significant differences in the frequencies of the three genotypes (GG, AG, and AA) among the longevity and two control groups (χ^2^ = 4.587, *p* = 0.318) for rs4420638 (Table 3).

The allelic frequencies of rs584007 and rs4420638 are shown in Table 4. The frequencies of the G and A alleles of rs584007 were 0.464 and 0.536, respectively. The frequencies of the G and A alleles of rs4420638 were 0.108 and 0.892, respectively.

### 3.4. Genotypes and Serum Lipid Levels

The levels of TC, TG, HDL-c, and LDL-c were not different among the three genotypes of the rs584007 in the three groups (*p* > 0.05). The levels of HDL-c for GG, AG, and AA were significantly different, while the levels of TG for AA and AG genotypes and the level of LDL-c for AG were significantly different (*p* < 0.05) among the three groups for rs584007. The levels of TG and HDL-c were significantly different among the three genotypes of rs4420638 in the longevity group (*p* < 0.05). The levels of TC for GG, AG and AA were significantly different in the Control 2 group, while the levels of TG and HDL-c for AA and AG genotypes were significantly different (*p* < 0.05) among the three groups for the rs4420638 (Table 5).

### 3.5. Risk Factors for Serum Lipid Parameters

Serum lipid parameters were associated with a few environmental factors, including age, gender, DBP, SBP, rs4420638, rs584007, and BMI (Table 6).

## 4. Discussion

In the present study, serum HDL-c and SBP levels in the longevity group were significantly higher than those in the two control groups, while serum TG and BMI in the longevity group were significantly lower than those in the two control groups. These characteristics are consistent with other geriatric studies [26,29]. The differences cannot completely be explained by the higher age of the long-lived populations.

Our study showed that the genotypic frequencies of rs584007 in diverse participants were different, while no significant difference was observed in allelic frequencies. Meanwhile, we observed no significant differences in the frequencies of the three genotypes (GG, AG, and AA) and allelic frequencies among the longevity and the two control groups for rs4420638. These results suggest that the prevalence of the ApoC-I rs584007 SNP may exhibit an age-related difference, while the prevalence of the ApoC-I rs4420638 SNP did not show such a difference. Our results are inconsistent with the results reported [12], which may be associated with the fact that the population was different, as different people have different genetic backgrounds. In our study, there were no significant differences in blood lipids among the three genotypes of the rs584007 within the group. The level of HDL-c was the highest in the longevity group and the lowest in the Control 2 group among the GG, AA, and AG genotypes, respectively, while this trend was the opposite of TG for AA and AG genotypes among the three groups. These were significantly different. The levels of TG and HDL-c were significantly different among the three genotypes of the rs4420638 in the longevity. The distributions of the levels of HDL-c and TG were similar to the rs584007 for AA and AG genotypes. Thus, carrying the A allic genotype was associated with the levels of HDL-c and TG.

In line with the findings of other researchers, the rise of HDL-c and the decline of TG can reduce cardiaccerebral vascular disease in the elderly [30,31,32]. Our data suggested that the rise in HDL-c and the decline in TG were due to rs584007 and rs4420638 SNPs of ApoC-I and environmental factors related to longevity. This may be because ApoC-I can regulate lipid metabolism and induce cardioprotection about the modulation of Δψm and oxidative phosphorylation [24].

The prevalence rates of some chronic diseases, such as hypertension, stroke, and diabetes, were lower in the longevity group than in the elderly [33]. Hyperlipidemia is one of the major risk factors for many chronic diseases in the elderly [34,35,36], whereas ApoC-I is involved as a cofactor in enzymatic reactions of lipid metabolism with high-density lipoproteins [3]. The lipid level’s relation to longevity is determined by multiple genetic and environmental factors, and the impact of one gene is very limited. It is speculated that the influence of ApoC-I on lipid metabolism may be limited, similar to other lipid-regulating genes such as phosphodiesterase 3A (PDE3A) rs7134375 [37], rs670 of the APOAI gene [38], and (cholesteryl-ester transfer protein) CETPTaq1B [39]. Although the effect of a sole gene can be small, multiple genes can produce larger effects. These effects can be influenced by environmental factors such as diet, lifestyle, and the interactions of other lipid-related genes and the environment via undetected pathways. In addition, people are contacted with different lifestyles and environments that can change the effects of the genetic variation on blood lipids.

We also observed that serum lipid parameters were correlated with age, gender, BMI, SBP, and rs4420638 in all participants. Our data showed that environmental factors also play an important role in the serum lipid levels of the longevity and two control populations. Diets and lifestyles were different in these different populations. Diets and lifestyles are similar to those described in our previous article [40]. Keeping this in mind, we can identify the genes related to blood lipids and suggest eating a healthy diet to reduce the impact of hyperlipidemia on people’s health.

However, some shortcomings of this study should be mentioned. Firstly, the sample size is a bit small. The results need to be further confirmed with larger sample sizes. Secondly, there is lack of information on life-behavior habits such as cigarette smoking and alcohol consumption. These may affect our results. Thirdly, we studied the association of genetic polymorphisms with serum lipid levels and human longevity, but there was only one gene. The longevity and blood lipids are influenced by multiple genes and environmental factors, so we should strengthen the study of the gene–gene and gene–environment interactions. HDL functionality has been recently proven to be more biologically relevant than HDL-c levels [41], and there are indeed reliable methods of determining HDL function [42]. In our paper, we did not determine HDL function, so we can study its function in the future.

## 5. Conclusions

The ApoC-I polymorphism might be one of the genetic factors of longevity in Bama. The ApoC-I rs4420638 and rs584007 SNPs are associated with serum TG and HDL-c levels in the longevous population.

## Figures and Tables

**Table 1:** A comparison of general characteristics and serum lipid levels between the longevity group and the two control groups.

Parameter	Longevity (*n* = 178)	Control 1 (*n* = 147)	Control 2 (*n* = 190)	χ^2^ (*F*)	*p*
Gender (m/f)	51/127	79/68	74/116	21.243	0.000
Age (year)	94.30 ± 4.21	65.14 ± 11.26	53.98 ± 10.51	909.417	0.000
SBP (mmHg)	144.35 ± 25.51	135.65 ± 23.43	121.49 ± 19.90	46.495	0.000
DBP (mmHg)	80.16 ± 12.94	79.70 ± 11.55	77.52 ± 12.13	2.418	0.090
BMI (kg/m^2^)	19.01 ± 2.77	20.40 ± 2.90	22.53 ± 3.30	63.843	0.000
TC (mmol/L)	4.76 ± 1.11	4.62 ± 0.87	4.87 ± 0.97	2.671	0.070
TG (mmol/L)	1.48 ± 0.96	1.67 ± 1.15	1.96 ± 0.94	10.702	0.000
HDL-c (mmol/L)	1.51 ± 0.76	1.35 ± 0.78	1.11 ± 0.25	18.432	0.000
LDL-c (mmol/L)	2.67 ± 0.80	2.60 ± 0.69	2.51 ± 0.60	2.626	0.073

Values are given as mean ± SDs. SBP: systolic blood pressure; DBP: diastolic blood pressure; BMI: body mass index; TC: serum total cholesterol; TG: serum total triglyceride; HDL-c: high-density lipoprotein cholesterol; LDL-c: low-density lipoprotein cholesterol.

**Table 2:** The chi-square test of Hardy–Weinberg equilibrium of the ApoC-I rs584007 and rs4420638.

Group	*n*	GG	AG	AA	χ^2^	*p*
Longevity	178	39	101	38	3.238	0.198
Control 1	147	35	62	50	3.202	0.202
Control 2	190	31	105	54	2.814	0.245
total (rs584007)	515	105	268	142	1.098	0.577
Longevity	178	3	27	148	1.717	0.424
Control 1	147	2	23	122	0.565	0.754
Control 2	190	4	43	143	0.130	0.937
total (rs4420638)	515	9	93	413	1.914	0.384

**Table 3:** Genotypic frequencies of the ApoC-I rs584007and rs4420638, *n* (%).

Group	*n*	Genotype *n* (%)			χ^2^	*p*
		GG	AG	AA		
Longevity	178	39 (21.9)	101 (56.7)	38 (21.4)	8.302 ^a^	0.016
Control 1	147	35 (23.8)	62 (42.2)	50 (34.0)	6.080 ^b^	0.048
Control 2	190	31 (16.3)	105 (55.3)	54 (28.4)	3.387 ^c^	0.184
Total (rs584007)	515	105 (20.4)	268 (52.0)	142 (27.6)	11.238 ^d^	0.024
Longevity	178	3 (1.7)	27 (15.2)	148 (83.1)	4.587	0.318
Control 1	147	2 (1.4)	23 (15.6)	122 (83.0)		
Control 2	190	4 (2.1)	43 (22.6)	143 (75.3)		
Total (rs4420638)	515	9 (1.7)	93 (18.1)	413 (80.2)		

^a^ Longevity vs. Control 1; ^b^ Control 1 vs. Control 2; ^c^ Longevity vs. Control 2; *p* < 0.017 indicates statistical significance; ^d^ total χ^2^ value.

**Table 4:** Distributions of alleles and MAF in ApoC-I rs584007 and rs4420638.

Group	G	A	MAF	χ^2^	*p*	OR	95% CI
	*n* (%)	*n* (%)					
Longevity	179 (50.3)	177 (49.7)	0.497	1.870	0.172 ^a^	0.806	0.591–1.098
Control 1	132 (44.9)	162 (55.1)	0.449	0.061	0.805 ^b^	0.962	0.708–1.307
Control 2	167 (43.9)	213 (56.1)	0.439	2.960	0.085 ^c^	0.775	0.580–1.036
total (rs584007)	478 (46.4)	552 (53.6)	0.464	3.342	0.188 ^d^	-	-
Longevity	33 (9.3)	323 (90.7)	0.093	0.001	0.970 ^a^	0.990	0.580–1.688
Control 1	27 (9.2)	267 (90.8)	0.092	2.908	0.088 ^b^	1.533	0.936–2.511
Control 2	51 (13.4)	329 (86.6)	0.134	3.133	0.077 ^c^	1.517	0.954–2.413
total (rs4420638)	111 (10.8)	919 (89.2)	0.108	4.380	0.112 ^d^	-	-

^a^ Longevity vs. Control 1; ^b^ Control 1 vs. Control 2; ^c^ Longevity vs. Control 2; *p* < 0.017 indicates statistical significance; ^d^ total χ^2^ value; OR: odds ratio; 95% CI: 95% confidence interval.

**Table 5:** Genotypes of the rs584007 and rs4420638 polymorphisms and serum lipid levels in the longevity and two control groups.

Genotypes	n	TC (mmol/L)	TG (mmol/L)	HDL-c (mmol/L)	LDL-c (mmol/L)
Longevity	rs584007				
GG	39	4.86 ± 1.30	1.67 ± 1.15	1.68 ± 1.02 *	2.73 ± 0.94
AG	101	4.64 ± 1.12	1.50 ± 0.94 *	1.49 ± 0.69 **	2.59 ± 0.82 *
AA	38	4.97 ± 0.83	1.21 ± 0.76 **	1.41 ± 0.62 *	2.85 ± 0.54
Control 1					
GG	35	4.48 ± 0.88	1.62 ± 1.00	1.26 ± 0.59	2.53 ± 0.65
AG	62	4.76 ± 0.86	1.77 ± 1.37	1.43 ± 0.97	2.72 ± 0.68
AA	50	4.55 ± 0.88	1.58 ± 0.96	1.31 ± 0.63	2.51 ± 0.72
Control 2					
GG	31	4.98 ± 0.97	1.95 ± 0.88	1.15 ± 0.26	2.48 ± 0.54
AG	105	4.84 ± 1.01	1.97 ± 1.06	1.09 ± 0.24	2.45 ± 0.61
AA	54	4.88 ± 0.92	1.92 ± 0.69	1.14 ± 0.28	2.64 ± 0.62
Longevity	rs4420638				
GG	3	4.99 ± 0.17	2.46 ± 1.70 *	2.37 ± 1.28 *	2.54 ± 0.17
AG	27	5.00 ± 1.26	1.74 ± 1.37 ^#^	1.65 ± 1.07 ^#^	2.90 ± 0.92
AA	148	4.71 ± 1.09	1.41 ± 0.84 ^##^	1.47 ± 0.67 ^##^	2.64 ± 0.78
Control 1					
GG	2	4.73 ± 1.52	2.00 ± 1.07	1.61 ± 1.06	2.21 ± 0.35
AG	23	4.53 ± 0.67	1.50 ± 0.66	1.20 ± 0.52	2.73 ± 0.58
AA	122	4.64 ± 0.90	1.70 ± 1.23	1.37 ± 0.82	2.58 ± 0.71
Control 2					
GG	4	5.36 ± 1.08 *	1.77 ± 0.53	1.14 ± 0.08	2.64 ± 0.59
AG	43	5.18 ± 1.15	2.17 ± 1.47	1.10 ± 0.22	2.67 ± 0.69
AA	143	4.77 ± 0.89	1.90 ± 0.71	1.12 ± 0.27	2.45 ± 0.57

** *p* < 0.01; * *p* < 0.05; ^#^ *p* < 0.05; ^##^ *p* < 0.01 (* three genotypes in a group; ^#^ a genotype among the three groups).

**Table 6:** Association between serum lipid parameters and relative factors in the participants.

Lipid Parameter	Risk Factor	Unstandardized Coefficient	Std. Error	Standardized Coefficient	t	*p*
All participants						
TC	SBP	0.005	0.002	0.134	2.234	0.026
	Gender	0.211	0.090	0.103	2.340	0.020
	rs4420638	−0.281	0.099	−0.127	−2.828	0.005
TG	Age	−0.008	0.003	−0.150	−2.891	0.004
	BMI	0.053	0.015	0.172	3.615	0.000
HDL-c	Age	0.004	0.002	0.126	2.362	0.019
	SBP	0.004	0.002	0.142	2.343	0.020
LDL-c	Gender	0.178	0.063	0.124	2.829	0.005
	rs4420638	-0.182	0.069	−0.117	−2.620	0.009
Longevity group and Control 1 group						
TC	Gender	0.345	0.121	0.168	2.859	0.005
	Age	−0.007	0.004	−0.122	−2.006	0.046
	SBP	0.010	0.003	0.258	3.840	0.000
TG	Age	−0.009	0.004	−0.147	−2.456	0.015
	BMI	0.086	0.020	0.237	4.246	0.000
HDL-c	BMI	0.045	0.015	0.171	2.946	0.003
LDL-c	Gender	0.285	0.091	0.186	3.132	0.002
	SBP	0.005	0.002	0.157	2.311	0.021
Longevity group						
TC	Gender	0.440	0.185	0.180	2.386	0.018
	SBP	0.013	0.004	0.309	3.667	0.000
	DBP	−0.015	0.007	−0.180	−2.148	0.033
TG	Gender	0.339	0.160	0.160	2.116	0.036
HDL-c	Gender	0.306	0.130	0.183	2.360	0.019
	rs4420638	−0.288	0.139	−0.163	−2.080	0.039
LDL-c	SBP	0.008	0.003	0.252	2.905	0.004
Control 1 group						
TC	Gender	0.389	0.141	0.223	2.763	0.007
	Age	−0.021	0.006	−0.272	−3.306	0.001
	Diastolic blood pressure	0.021	0.008	0.277	2.638	0.009
	BMI	0.067	0.024	0.223	2.794	0.006
TG	BMI	0.134	0.033	0.338	4.111	0.000
HDL-c	Age	−0.016	0.006	−0.227	−2.734	0.007
	BMI	0.094	0.022	0.350	4.340	0.000
	Gender	0.418	0.118	0.304	3.536	0.001
	Diastolic blood pressure	0.013	0.007	0.224	2.002	0.047
Control 2 group						
TC	SBP	−0.017	0.005	−0.339	−3.118	0.002
	DBP	0.027	0.009	0.334	3.138	0.002
	Age	0.019	0.007	0.201	2.807	0.006
	rs4420638	−0.419	0.141	−0.211	−2.980	0.003
	BMI	0.045	0.022	0.153	2.035	0.043
TG	Gender	−0.342	0.142	−0.178	−2.414	0.017
HDL-c	Gender	0.105	0.038	0.201	2.746	0.007
	Age	0.004	0.002	0.154	2.051	0.042
	DBP	0.005	0.002	0.236	2.118	0.036
LDL-c	SBP	−0.010	0.003	−0.317	−3.103	0.002
	DBP	0.016	0.005	0.323	3.227	0.001
	Age	0.015	0.004	0.264	3.931	0.000
	rs584007	0.133	0.060	0.145	2.196	0.029
	rs4420638	−0.272	0.082	−0.220	−3.312	0.001
	BMI	0.060	0.013	0.327	4.646	0.000
